# Development of Microsatellite Markers from Transcriptome of *Eriocheir sinensis* and Their Application in Multiplex PCR Panels

**DOI:** 10.3390/ani14223200

**Published:** 2024-11-08

**Authors:** Qizhen Xiao, Meijun Tang, Qingqing Li, Pengsheng Dong, Yongxu Cheng, Deng Deng, Xugan Wu

**Affiliations:** 1 Key Laboratory of Freshwater Aquatic Genetic Resources, Ministry of Agriculture, Shanghai Ocean University, Shanghai 201306, China; xiaoqizhen@stu.xmu.edu.cn (Q.X.); mjtang@shou.edu.cn (M.T.); liqingqing@zhku.edu.cn (Q.L.); dpsh@foxmail.com (P.D.); yxcheng@shou.edu.cn (Y.C.); 2 Fujian Provincial Key Lab of Coastal Basin Environment, Fujian Polytechnic Normal University, Fuqing 350300, China; 3 Dongying Huize Agricultural Technology Co., Ltd., Dongying 257503, China; 4 Shanghai Collaborative Innovation Center for Aquatic Animal Genetics and Breeding, Shanghai Ocean University, Shanghai 201306, China; 5 National Demonstration Center for Experimental Fisheries Science Education, Shanghai Ocean University, Shanghai 201306, China; 6 Shenzhen Alpha Feed Co., Ltd., Shenzhen 518054, China

**Keywords:** SSR, Chinese mitten crab, multiplex PCR, family selection, genetic diversity, parentage assignment

## Abstract

Background: The development of genetic markers is crucial for aquaculture and population genetics. Polyallelic microsatellite markers are widely used for breeding and family management of *Eriocheir sinensis* (*E. sinensis*), contributing to the sustainable development of the industry. Methods: Through transcriptome analysis, 100 highly polymorphic SSR markers were identified and developed. From these, four multiplex PCR panels, each containing 16 markers, were constructed and applied to parentage assignment in six *E. sinensis* families. Results: The number of alleles (*N_a_*) ranged from 7 to 21, the observed heterozygosity (*H_o_*) varied from 0.356 to 0.951, and the expected heterozygosity (*H_e_*) varied from 0.656 to 0.909. The polymorphism information content (*PIC*) ranged from 0.633 to 0.900. The CERVUS analysis showed that using only the two most informative multiplex PCR panels, assignment success rates exceeded 90.34%, with 100% accuracy in assigning offspring to parents when three or four panels were used. Conclusions: This method significantly enhances the efficiency of microsatellite marker analysis, which provides a convenient and cost-effective method for breeding programs and family management of *E. sinensis*.

## Simple Summary

As a key economic species in China, the breeding of new strains of Chinese mitten crab (*Eriocheir sinensis*) has been undertaken across various regions to address genetic resource degradation caused by the industry’s rapid expansion. Limited pond space often resulted in different families of *Eriocheir sinensis* being raised together, complicating the preservation of distinct family lines. Microsatellite markers are widely used to assess the genetic diversity and assign parentage, but they can be expensive to develop and apply. Advances in high-throughput sequencing technology have made these markers more accessible, while multiplex PCR can significantly reduce costs. In this study, 100 highly polymorphic SSR markers were developed from the transcriptome, four multiplex PCR panels were constructed, and a parentage assignment method was created for *Eriocheir sinensis*. The results demonstrated that 100% of offspring were accurately assigned to their parents across six crab families when three or four panels were used. This method provides an efficient and cost-effective solution for breeding and family management, promoting the sustainable development of the industry.

## 1. Introduction

The Chinese mitten crab, *Eriocheir sinensis* (*E. sinensis*), a member of the Grapsidae family of decapod crustaceans, is widely distributed in the East Asian–Pacific region and holds significant aquaculture value in China. Due to its high nutritional content and market demand, *E. sinensis* is extensively cultured throughout China, with a production volume reaching 888,629 tons in 2023 [1]. The cultivation of *E. sinensis* supports a vital industry in eastern China, attracting increasing numbers of farmers. However, this expansion has introduced challenges such as sexual precocity [2,3], hepatopancreatic necrosis disease [4], inbreeding, and the degeneration of genetic resources [5]. These issues arise largely because many farmers rely on small-scale stock for breeding or indiscriminately hybridize geographically distinct wild populations with poor growth traits or lower taste quality in an attempt to reduce costs. Consequently, the genetic quality and overall performance of aquaculture stocks have deteriorated. To address these challenges, it is crucial to protect the genetic resources of *E. sinensis* and guide the industry towards more sustainable breeding practices. Family-based selection, which has proven effective in the breeding of plants and livestock, holds great potential for aquaculture as well [6]. By constructing large-scale families, breeders can accurately trace genealogical information, select high-quality parents, and avoid inbreeding. This approach also helps to continuously improve desirable traits and shortens the breeding cycle [6,7]. While family-based selection offers many benefits, it has not been widely adopted in *E. sinensis* breeding due to the high costs associated with maintaining large family groups. Assigning each family to a specific pond can help minimize environmental variations and reduce the costs of maintaining separate family lines [8]. Traditionally, physical tags have been used to distinguish individual lineages [9,10]. However, applying physical tags to *E. sinensis* is problematic, as the crabs molt frequently, making it difficult to attach and maintain tags on their bodies. Implanting electronic tags may offer a solution, but the crabs need to reach a sufficient size, and the technology for reliable tagging is still under development. Tags are prone to falling off, and the mortality rate may increase if the tags are improperly applied. A more effective solution is to house different families in the same pond and use DNA molecular markers to distinguish between them.

Molecular markers such as random amplification polymorphism DNA (RAPD), amplified fragment length polymorphism (AFLP), microsatellites (also known as simple sequence repeat, SSR), and single-nucleotide polymorphisms (SNPs) are widely used in breeding programs [11,12]. While SNPs have been increasingly applied in genetic studies of crustaceans such as Pacific white shrimp (*Litopenaeus vannamei*) [13,14], mud crab (*Scylla paramamosain*) [15], and swimming crab (*Portunus trituberculatus*) [16], their development and analysis require more advanced and costly technologies. In contrast, SSR markers remain a preferred tool for genetic diversity analysis [17,18] and parentage identification [19,20] due to their accessibility. Microsatellite markers have been widely utilized for over a decade due to their high abundance, variability, co-dominant inheritance, and neutral selection within the genome. They typically consist of tandem repeating units of mono-, di-, tri-, and tetra-nucleotide sequences [21,22]. Their high allelic diversity provides superior power for determining pedigree relationships among individuals [23]. In *E. sinensis*, microsatellite markers are commonly used for constructing linkage maps [24,25], assessing population genetic diversity [26,27,28], and conserving and exploiting wild crab genetic resources [29,30]. For family management, the use of microsatellite markers in *E. sinensis* offers several advantages, including reduced maintenance costs, savings in manpower and space, and a more reliable method for evaluating genetic parameters. However, despite these benefits, the cost of genotyping large numbers of individuals with microsatellite markers can still be considerable.

Establishing microsatellite multiplex PCR panels is useful for evaluating parentage assignment in applied fisheries, particularly when managing large numbers of families. Multiplex PCR panels reduce the time and cost associated with genetic diversity evaluations using SSR markers, minimizing repetitive manipulations and manual handling errors [31]. These panels have been successfully implemented in several aquatic species, including grass carp (*Ctenopharyngodon idellus*) [32], mussel *Anodonta* (*Sinanodonta*) *woodiana* (Lea, 1834) [33], and swimming crab (*Portunus trituberculatus*) [34]. However, research on the development of multiplex PCR systems for *E. sinensis* remains limited, despite their potential to enhance large-scale family breeding programs.

With the advent of next-generation sequencing technology for transcriptomics, the acquisition of a substantial number of SSR markers has become more cost-effective. In this study, we sequenced the transcriptomics of *E. sinensis*, identifying 1,521,519 putative microsatellite markers. From 272 designed SSR primer pairs, 205 were validated, with 100 demonstrating high polymorphism. By screening these polymorphic microsatellite markers, we developed four microsatellite multiplex PCR panels for the first time in *E. sinensis* and applied them to determine parentage in six families. This approach significantly enhances analytical efficiency and offers a practical and effective method for breeding and family management of *E. sinensis*, promoting the sustainable development of the industry.

## 2. Materials and Methods

### 2.1. Experimental Animals and Tissue Collection

Samples for transcriptome sequencing were collected at the Chongming Research Station of Shanghai Ocean University (N 31°37′, E 121°23′), where continuous breeding and lineage establishment of *E. sinensis* has been conducted for an extended period. Based on previous research on ovarian development [35], we sampled three successive developmental stages (Stages I, II, and III) of *E. sinensis*, collecting three ovarian tissue samples from each stage. These samples were promptly stored in liquid nitrogen for subsequent analysis. For polymorphism validation of putative SSR markers and the construction of multiplex PCR panels, samples were obtained from 15 wild crabs collected from Zhenjiang, Jiangsu province (N 32°11′, E 119°25′), and 15 pond-cultured crabs from Chongming district, Shanghai City (N 31°37′, E 121°23′). Additionally, samples for multiplex PCR construction and parentage assignment were obtained from six families, consisting of 12 adult parents and 207 offspring (Table 1). The third appendage muscle from each sample was collected and preserved in 95% ethanol for DNA analysis. Total genomic DNA was extracted using a modified phenol–chloroform method [36]. The quantity and purity of the extracted DNA were assessed with a Q5000 UV spectrophotometer (Qwawell, San Jose, CA, USA), and DNA quality was confirmed through 1% agarose gel electrophoresis. Total RNA was extracted from the ovarian tissues using the TRIzol@ Reagent (TaKaRa, Kyoto, Japan) following the manufacturer’s protocol. The quantity and quality of total RNA were measured using a Q5000 UV spectrophotometer (Qwawell, USA) and a 2100 Bioanalyzer (Agilent Technologies, Santa Clara, CA, USA), respectively.

### 2.2. Transcriptome Sequencing, Assembly, and Microsatellite Identification

Transcriptome sequencing was conducted using the Illumina HiSeq™ 2500 platform (Illumina, Inc., San Diego, CA, USA) [37]. After filtering out reads containing adapters, poly-N sequences, and low-quality reads, clean reads were aligned to the reference genome assembly using the HISAT v2.0.4 program [38]. Microsatellite markers with a 10 bp minimum repeat length, encompassing di- to hexa-nucleotide motifs, were identified using MISA software 1.0, resulting in the identification of 1,521,519 putative SSR markers. Subsequently, primers were designed for these markers using Primer 5.0 [39], leading to the selection of 272 SSR markers for polymorphism evaluation. Each marker was PCR-amplified individually in 30 samples. The PCR amplification was carried out in a final volume of 12.5 µL, containing 6.25 µL of Premix Taq (which include 0.5U ExTaq, 0.4 mM dNTP mixture, and 4 Mm EXTaq buffer, Novoprotein Biotech Co., Ltd., Suzhou, China), 0.5 µL of each primer (at a concentration of 10 pmol/µL), 1 µL of template DNA (approximately 50 ng), and 4.25 µL of double-distilled water. One primer from each pair was labeled with the fluorescent dye HEX or FAM (see Table 2 for details). The primers were synthesized by Sangon Biotech (Shanghai Co., Ltd., Shanghai, China). The PCR conditions included an initial denaturation step at 94 °C for 5 min, followed by 35 cycles of 30 s at 94 °C, 30 s at the optimal annealing temperature (as indicated in Supplementary Table S1), and 1 min at 72 °C, concluding with a final extension step at 72 °C for 10 min. The quality of the PCR products was assessed using 1% agarose gel electrophoresis, and all PCR products were analyzed on an automatic capillary sequencer Applied Biosystems 3730XL (Thermo Fisher Scientific, Waltham, MA, USA) using the GeneScan 500 LIZ size standard (Thermo Fisher Scientific, Waltham, MA, USA). The polymorphic microsatellite markers were retained to construct multiplex PCR panels.

### 2.3. Multiplex PCR Panel Optimization

The polymorphic microsatellite markers were combined into multiplex PCR reactions using Multiplex Manager1.0 software [40] to evaluate potential issues such as mismatches, primer dimers, and hairpin structures. We tested the SSR multiplex PCR panels using 30 samples of *E. sinensis*. The multiplex PCR amplifications were conducted in a final volume of 12.5 µL, containing 50 ng of template DNA, 6.25 µL of Premix Taq (as detailed in Section 2.2), and 0.5 µL of each primer (as detailed in Section 2.2), with double-distilled water added to achieve the total volume. The thermal cycling protocol consisted of an initial denaturation at 94 °C for 3 min, followed by 35 cycles of 94 °C for 1 min, 55 °C for 1 min, 72 °C for 1 min, and a final extension step at 72 °C for 10 min. To optimize amplification and ensure comparable fluorescent signals, the volume of primers was adjusted from the initial volume of 0.05 µL. The PCR products were analyzed on an automatic capillary sequencer Applied Biosystems 3730XL (Thermo Fisher Scientific, Waltham, MA, USA) with the GeneScan 500 LIZ size standard (Thermo Fisher Scientific, Waltham, MA, USA).

### 2.4. Parentage Assignment

The SSR multiplex PCR panels were utilized for parentage assignment in six families of *E. sinensis*, with the number of progeny assigned to each family detailed in Table 1. Cervus v3.0 [41] was used for parentage analysis based on the genotyping data with the maximum likelihood method. This analysis included both simulated and actual identification rates, with a default genotyping error rate set at 1% and confidence intervals at 95%. Using genotype data from the offspring and candidate parents, the parentage assignment module determined the parental pair for each offspring.

### 2.5. Data Analysis

Alleles were assigned by GeneMapper Software v4.0 (Applied Biosystems) and potential genotyping errors were checked with Micro-Checker Software 2.2.3 [42]. The following genetic parameters were calculated using POPGENE 3.2 [43]: number of alleles (*N_a_*), number of effective alleles (*N_e_*), observed heterozygosity (*H_o_*), expected heterozygosity (*H_e_*), Shannon index (*I*), and departure from the Hardy–Weinberg equilibrium (*HW*). Polymorphism information content (*PIC*) was calculated using PICcalc 0.6 [44]. Parentage assignments were performed using CERVUS 3.0 software [41].

## 3. Results

### 3.1. Primer Design and Validation

**Figure 1:**
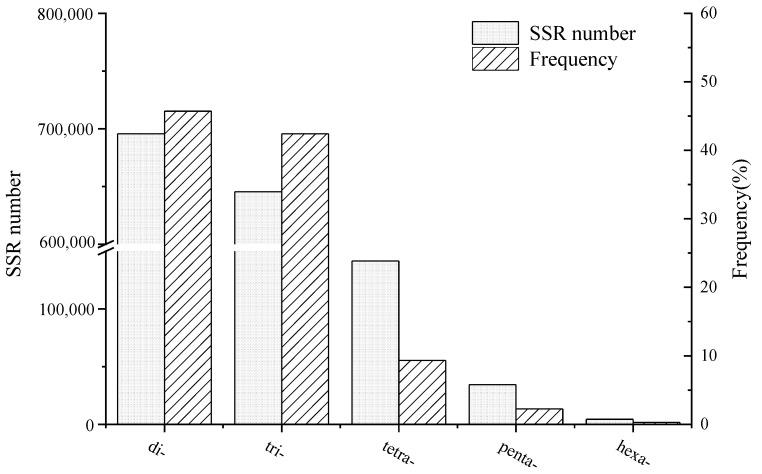
Distribution of different SSR types in *Eriocheir sinensis* transcriptome. A total of 1,521,519 putative SSRs were identified from the transcriptome data of *E. sinensis*, with 45.72% being di-nucleotide repeats, 42.42% tri-nucleotide repeats, 9.31% tetra-nucleotide repeats, 2.26% penta-nucleotide repeats, and 0.29% hexa-nucleotide repeats.

A total of 1,521,519 putative SSR markers were identified from the transcriptome data of E. sinensis (Figure 1). Among these, 695,674 SSR markers were di-nucleotide repeats (45.72%), 645,399 SSR markers were tri-nucleotide repeats (42.42%), 141,615 SSR markers were tetra-nucleotide repeats (9.31%), 34,445 SSR markers were penta-nucleotide repeats (2.26%), and 4386 SSR markers were hexa-nucleotide repeats (0.29%) (Figure 1). To ensure unbiased and specific selection of SSR markers, 272 SSR markers were randomly chosen for polymorphism assessment in 30 crabs (Table 3). The results demonstrated that 205 of the selected markers produced clear and stable bands within the expected fragment range, with 100 markers exhibiting polymorphisms (Table 3 and Supplementary Table S1).

### 3.2. Established SSR Multiplex PCR Panels

**Figure 2:**
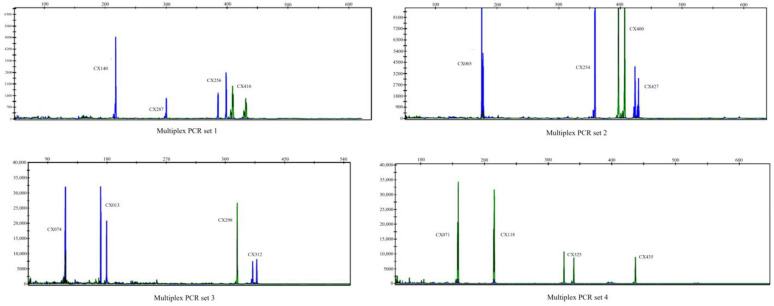
Part of gene scan results of four sets of multiplex PCR in *Eriocheir sinensis*. The blue peak is FAM fluorescent dye and the green peak is HEX fluorescent dye.

The same panel of 30 crabs was used for the optimization of multiplex PCR systems. Four sets of optimized multiplex PCR panels were developed based on primer annealing temperatures, allelic size ranges, and compatibility of fluorescent labeling dyes. Each set consisted of four markers as follows: panel 1 included *CX140*, *CX287*, *CX256*, and *CX416*; panel 2 contained *CX003*, *CX254*, *CX400*, and *CX427*; panel 3 comprised *CX074*, *CX013*, *CX298*, and *CX312*; and panel 4 featured *CX071*, *CX118*, *CX325*, and *CX435* (Table 2). The partial gene scans for the four multiplex PCR panels in *E. sinensis* are depicted in Figure 2, demonstrating that the primer amplification for each set of multiplex PCR reactions produced similar fluorescence signal peaks. The use of two fluorescent dyes, 6-FAM and HEX, facilitated the overlap of similarly sized markers, enabling the successful amplification of all markers across the different panels. To verify the feasibility of multiplex PCR in assessing genetic diversity, these 16 markers were subsequently evaluated in 207 individuals. All markers exhibited polymorphism, with the number of alleles (*N_a_*) ranging from 7 to 21. The observed heterozygosity (Ho) varied from 0.356 to 0.951, while the expected heterozygosity (He) ranged from 0.656 to 0.909 (Table 4). Furthermore, the polymorphism information content (*PIC*) ranged from 0.633 to 0.900, and it was found that 13 markers deviated from the Hardy–Weinberg equilibrium (*p* < 0.01) (Table 4).

### 3.3. Result of Parentage Assignment

**Figure 3:**
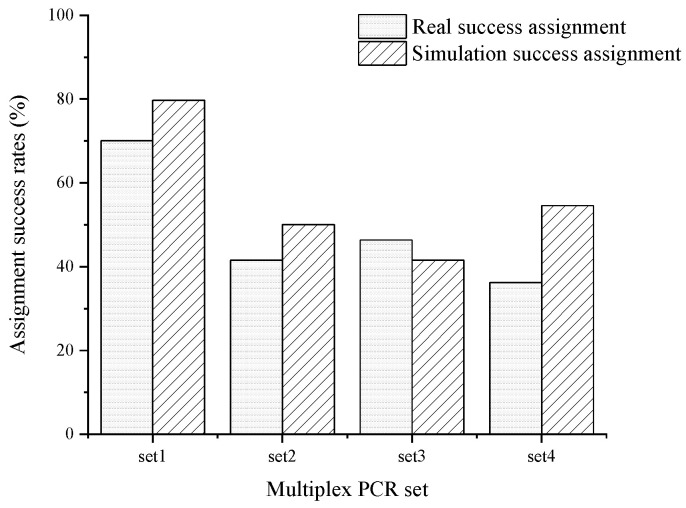
The real success assignment rate and simulation success assignment rate of four sets of multiplex PCR. The actual identification rates were 70.05%, 41.55%, 46.38%, and 36.23% for sets 1, 2, 3, and 4, respectively, while simulated rates were 79.71%, 50.07%, 41.55%, and 54.59%.

Parentage assignments for six families were analyzed using microsatellite multiplex PCR in conjunction with the maximum likelihood method via CERVUS 3.0 [41]. The actual identification rates for the known parents were 70.05%, 41.55%, 46.38%, and 36.23% for multiplex PCR sets 1, 2, 3, and 4, respectively. Simulation results indicated assignment success rates of 79.71%, 50.07%, 41.55%, and 54.59% for sets 1, 2, 3, and 4, respectively (Figure 3). Notably, the simulated identification rates were generally higher than the actual rates, with the exception of multiplex PCR set 3.

**Figure 4:**
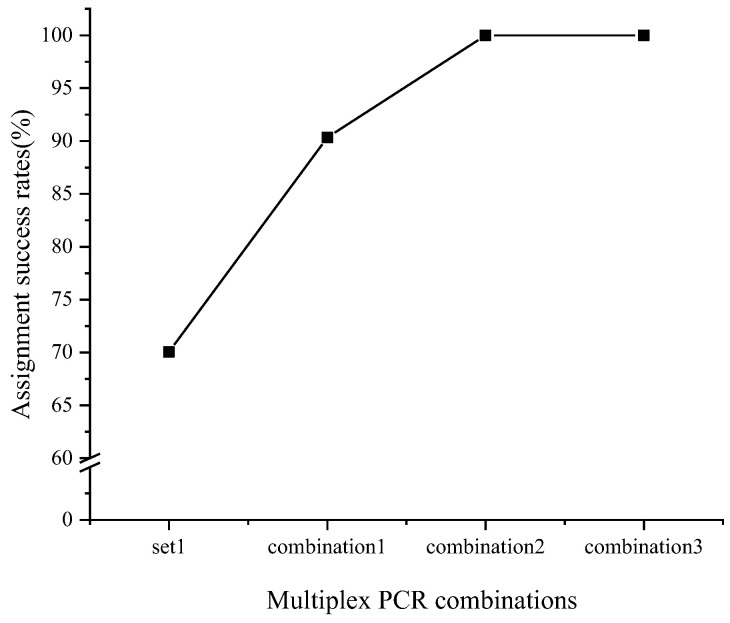
Assignment success rate of *Eriocheir sinensis* with different combinations of multiplex PCR panels. Combination 1 is set 1 + set 3, combination 2 is set 1 + set 3 + set 2, and combination 3 is set 1 + set 3 + set 2 + set 4. At a 95% confidence level, using the two highest-performing multiplex PCR sets (set 1 and set 3) achieved assignment accuracy rates of over 90.34%, while using three or four sets resulted in 100% correct offspring–parent allocation.

At a 95% confidence level, cumulative parentage assignment was prioritized based on the actual identification rates of each multiplex PCR system, arranged from highest to lowest. The results demonstrated that employing the two multiplex PCRs (set 1 and set 3) achieved assignment accuracy rates exceeding 90.34% (Figure 4). Furthermore, the use of three or four multiplex PCR sets resulted in 100% correct allocation of offspring to their respective parents (Figure 4).

## 4. Discussion

With the advent of next-generation sequencing technology, the discovery of SSR markers is no longer confined to constructing SSR libraries and sequencing candidate clones. Given the challenges of obtaining high-quality genome-wide information for non-model organisms, an increasing number of studies have shown that high-throughput microsatellite marker development from transcriptome data is an effective approach [45]. In this study, we conducted transcriptome sequencing of the ovaries of *Eriocheir sinensis* at different developmental stages using the Illumina HiSeq™ 2500 platform, resulting in the identification of 1,521,519 candidate microsatellite markers. Among these SSR markers, di-nucleotide repeat motifs were the most prevalent (45.72%), followed closely by tri-nucleotide repeat motifs (42.42%). These findings align with previous reports on *E. sinensis*, which indicated that di-nucleotide repeat motifs were the most abundant type (58.54%), followed by tri-nucleotide repeats (30.11%) [28]. It is generally recognized that most SSR repeats in animals are di-nucleotide repeats [46,47], and our results support this observation. To validate the identified SSR markers, we randomly selected a subset of 272 primer pairs for PCR, achieving a high polymorphism rate of 48.8% (100 out of 205) for the SSR markers. The lower polymorphism rates observed in our study compared to previous reports on *E. sinensis* may stem from false positives inherent in transcriptome sequencing, as well as variations in SSR search tools and criteria employed [48]. The number of SSR markers found in the transcriptome was lower, which is expected since transcriptome sequences primarily represent functional genes with fewer repeat sequences in coding regions; most repeated sequences are typically found in the untranslated regions (UTRs). Additionally, the challenge of amplifying larger intron regions can hinder the efficiency of microsatellite development in the transcriptome, necessitating the selection of primers that amplify smaller fragments. However, microsatellite markers developed from the transcriptome are often derived from regulatory or coding regions, enabling the direct and accurate tagging of functional genes, which is beneficial for studying functional variation, adaptive evolution, and genetic diversity in species during genetic processes [49,50]. In summary, this transcriptome provides a valuable resource for future gene analyses, and these microsatellite markers will be instrumental in advancing our understanding of the molecular ecology and genetic breeding of *E. sinensis*.

The establishment of microsatellite multiplex PCR represents the most efficient method for evaluating parentage assignment in applied fisheries, especially when a large number of family separations is required. This study is the first to establish a microsatellite multiplex PCR panel for *E. sinensis*. We developed four multiplex panels, each containing four microsatellite markers, designed based on allelic size range and compatibility of fluorescent labeling dyes. A critical factor in establishing and applying a multiplex microsatellite assay is the appropriate selection of sites and their combinations [51]. The concentration of each primer in the multiplex PCR reaction system is particularly crucial, albeit a tedious and challenging step. Various factors influence primer concentrations, including annealing temperature, nucleotide sequence patterns, and PCR product size [34,52]. Research suggests that the size difference between fragments at two markers should exceed 20 bp to minimize potential biases in amplification [53]. In our assay, the product fragment sizes between markers were greater than 20 bp or labeled with different fluorescent markers, allowing for accurate allele peak distinction. Ultimately, we successfully developed four SSR multiplex PCR sets for *E. sinensis*, which can serve as effective tools for genetic studies, including population genetic structure assessments and parentage assignments.

Beyond *E*. *sinensis*, multiplex PCR has been widely applied in other aquatic species. For instance, in the giant freshwater prawn (*Macrobrachium rosenbergii*), multiplex PCR has been utilized for genetic analysis and parentage verification. Similarly to our findings, studies on *Macrobrachium rosenbergii* have emphasized the efficiency of multiplex PCR in reducing time and reagent costs while maintaining high accuracy in genetic assessments. However, it is noteworthy that genetic diversity in *Macrobrachium rosenbergii* populations tends to be lower than that in *E. sinensis* [54,55]. In grass carp (*Ctenopharyngodon idellus*), multiplex PCR has been successfully applied to evaluate genetic diversity in breeding programs, revealing higher genetic diversity in cultured populations compared to *E. sinensis,* with a mean Na of 22.15, mean Ho of 0.823, mean He of 0.859, and mean PIC of 0.846 across nine populations [32]. The successful application of multiplex PCR across various aquatic animals underscores its value as a versatile and powerful tool for genetic analysis and breeding management.

Microsatellite markers have been extensively used for parentage assignment in various aquatic animals, such as orange-spotted grouper (*Epinephelus coioides*) [56], tiger trout (*Salmo trutta* × *Salvelinus fontinalis*) [57], and red swamp crayfish (*Procambarus clarkii*) [58]. These markers have significantly reduced the workload associated with distinguishing different families compared to traditional physical tagging methods. In our study, parentage assignment was performed on six families using microsatellite multiplex PCR, achieving 100% accuracy in assigning offspring to their parents when three or four multiplex PCR sets were utilized. Varying success rates for parentage assignments have been reported previously; for example, Zhang et al. (2016) used nine SSR markers to identify 18 full-sib families in yellow catfish *Pelteobagrus Fulvidraco*, achieving 100% allocation for all progeny [59]. Similarly, Satyanarayana et al. (2015) achieved a 91% assignment success rate using four SSR markers in freshwater prawn *Macrobrachium rosenbergii* [60]. Yang et al. (2014) reported a 97% success rate with ten SSR markers in mandarin fish *Siniperca chuatsi* [61]. The success rate observed in our study meets the practical needs for production applications.

Our findings suggest that the accuracy of parentage assignment depends on the number of markers used and their polymorphism; a lower-locus polymorphism necessitates a higher number of markers for reliable assignment. Previous studies have emphasized that selecting microsatellite markers with high polymorphism enhances the power of parentage assignment [59,62]. Moreover, the ability to identify paternity diminishes as the number of candidate parents increases, underscoring the need for an increased number of molecular markers alongside the growing number of parents and offspring. For instance, it was shown that with the same number of microsatellite markers, identification rates were 92.2% with three pairs of candidate parents but only 78.9% with eight pairs [63]. While higher identification rates can be achieved with a greater number of microsatellite markers, this approach also increases workload and optimal number of markers. In our study, 100% of the offspring were accurately allocated to their parents when using 12 microsatellite markers.

Additionally, our results indicate that the simulated assignment success rates were higher than the actual assignment rates. This discrepancy may be attributed to genotyping errors and the presence of null alleles, which are known to impact the accuracy of parentage assignments [64]. Such issues could arise from mutations or indels at one or both of the primer-binding sites [65]. Similar phenomena have been reported in studies involving the pacific oyster *Crassostrea gigas* [66] and yellow catfish [59]. Notably, some markers (*CX140*, *CX416*, *CX003*) exhibited high null allele frequencies; however, the error rates in parentage assignments can be mitigated by utilizing a combination of all markers, enabling the correct identification of offspring and assignment to their most likely parent.

## 5. Conclusions

In conclusion, this study successfully developed 100 polymorphic SSR markers from the transcriptome and established four SSR multiplex PCR sets for parentage assignment in *E. sinensis*. This method significantly enhances analytical efficiency and provides a convenient and effective approach for breeding and family management, thereby promoting the sustainable development of the industry. However, potential limitations should be considered, particularly in large-scale breeding programs. One notable challenge is the cost associated with maintaining and optimizing these multiplex panels when genotyping a substantial number of individuals. Additionally, as the number of markers or individuals increases, issues such as unequal amplification efficiency and cross-amplification between markers may arise. To mitigate these challenges, integrating multiplex PCR with advanced technologies, such as next-generation sequencing, or automating PCR setups could help enhance scalability and reduce labor costs. Despite these limitations, multiplex PCR remains a valuable tool for genetic analysis, particularly for targeted breeding and population management in *E. sinensis*.

## Figures and Tables

**Table 1:** The number of progeny assigned to six families of *Eriocheir sinensis*.

Families	Number of Females	Number of Males
C1	8	4
C2	15	15
C3	13	25
C4	14	25
C5	15	15
C6	41	17

**Table 2:** Characteristics of the four sets of multiplex PCR panels of microsatellites in *Eriocheir sinensis*.

Locus	Repeat Motif	Primer Sequences (5′−3′) ^a^	Annealing Temperature	Size Range (bp)	Concentration in Multiplex-PCR (µmol·L^−1^)	(GenBanK Accession No.)
PCR multiplex set 1			60 (°C)			
*CX140*	(CAA)_4_	F: FAM-TCACCACCATCAGCAACA		196–223	0.2	MF686621
		R: AGAAAGGACAGGGATAACC				
*CX287*	(GGC)_5_	F: FAM-TCACTAGGATAGGTAACGGA		292–327	0.4	MF686652
		R: CACTCAACACACGACAGGA				
*CX256*	(CCT)_4_	F: HEX-ATGGCTCTCTCTGGTGGCT		359–429	0.28	MF686639
		R: TGCTGAAATCGGAAGGTT				
*CX416*	(TGG)_4_	F: FAM-GTAGTGTGAAGCAAGGAGGA		381–445	0.36	MF686673
		R: GGAGTTTGAGGTGAGCGA				
PCR multiplex set 2			60 (°C)			
*CX003*	(TATT)_4_	F: FAM-CCCTCAACCTGAACCTCT		119–187	0.2	MF686585
		R: ACTCTTTTTTGCTGCCCT				
*CX254*	(TCC)_9_	F: FAM-CTATCCATCTCTCTACCTCCC		357–374	0.24	MF686638
		R: GCAAAAACAAACCACTAACC				
*CX400*	(GAG)_7_	F: HEX-ACCCTATCAAACTATTACGACC		401–411	0.24	MF754123
		R: ATGAACACCGAGACCACG				
*CX427*	(GTG)_4_	F: FAM-GGCATTCAATCTTAAACCC		415–435	0.36	MF686679
		R: ATCTCCTCCTTCCCTCGT				
PCR multiplex set 3			55 (°C)			
*CX074*	(CA)_6_	F: FAM-GCAAAACTTGGCAAACGA		110–119	0.2	MF686606
		R: ACCTCCACCCAGCACACT				
*CX013*	(GGA)_4_	F: FAM-ACTACGACTTTTCTACTTACCC		167–179	0.2	MF686590
		R: CCTCCTTATCCTCTTGCTC				
*CX298*	(CAC)_4_	F: HEX-ACCCAAGCACACCTTCTCC		332–381	0.2	MF686657
		R: CCAACCCTCCCAACCTAAT				
*CX312*	(TGG)_6_	F: FAM-ATCACCAACACTGCTCTCG		399–411	0.24	MF686661
		R: CTCAATGCTGCTGCCTCT				
PCR multiplex set 4			55 (°C)			
*CX071*	(TGG)_8_	F: HEX-GCTGGAGTCAGGGCTAAA		148–171	0.24	MF686604
		R: CAAAACACACACGGAAGAA				
*CX118*	(AAG)_4_	F: HEX-CCACAACTGCTGCTGATG		214–264	0.2	MF686612
		R: TGTAGACGAAGGAAGGCT				
*CX325*	(CTC)_5_	F: HEX-GAGCGATTAGTTACGGGA		322–357	0.4	MF686664
		R: GAGGAAGGTGTGTTTGGA				
*CX435*	(CTC)_5_	F: HEX-CATCATCTCCATCTCCTCTG		405–447	0.4	MF686683
		R: ATCTGCACCCCTTCTCAC				

Note: ‘a’ denotes the primer direction from 5′ to 3′ end.

**Table 3:** The universal and polymorphic potential of the 272 microsatellite markers in *Eriocheir sinensis*.

Type of SSR	Number of SSR	Universality		Polymorphism	
		Number	Probability (%)	Number	Probability (%)
Di-nucleotide	5	3	60.0	3	100
Tri-nucleotide	259	197	76.1	93	47.2
Tetra-nucleotide	4	3	75.0	3	100
Penta-nucleotide	3	1	33.3	0	0.0
Hexa-nucleotide	1	1	100	1	100
Total	272	205	75.4	100	48.8

**Table 4:** Characteristics of the 16 microsatellite markers in *Eriocheir sinensis*.

Locus	*N_a_*	*H_o_*	*H_e_*	*PIC*	*HW*	*F*(Null)
*CX140*	15	0.435	0.724	0.697	***	0.2385
*CX287*	9	0.822	0.867	0.850	***	0.0261
*CX256*	21	0.935	0.909	0.900	NS	−0.0182
*CX416*	10	0.488	0.767	0.736	***	0.2296
*CX003*	8	0.356	0.758	0.731	***	0.3725
*CX254*	15	0.877	0.852	0.833	***	−0.0179
*CX400*	9	0.849	0.792	0.762	***	−0.0389
*CX427*	9	0.617	0.838	0.816	***	0.1503
*CX074*	9	0.621	0.858	0.840	***	0.1583
*CX013*	7	0.656	0.810	0.785	***	0.0957
*CX298*	7	0.660	0.778	0.746	***	0.0876
*CX312*	7	0.757	0.808	0.780	***	0.0395
*CX071*	9	0.626	0.734	0.701	***	0.0928
*CX118*	11	0.587	0.656	0.633	NS	0.0334
*CX325*	16	0.951	0.898	0.887	NS	−0.0314
*CX435*	11	0.681	0.829	0.804	**	0.0929
Mean value	10.81	0.682	0.805	0.781		0.0944

Note: *N_a_*, number of alleles; *H_o_*, observed heterozygosity; *H_e_*, expected heterozygosity; *PIC*, polymorphism information content; *HW*, Hardy–Weinberg equilibrium *p* value, NS: nonsignificant. All the *p* values of deviations from the HW equilibrium marked by asterisks are significant; significant deviations at ** *p* < 0.01, *** *p* < 0.001. *F*(Null), the null allele frequency.

## Data Availability

The transcriptomic raw data were uploaded to the National Center for Biotechnology Information (NCBI) (accession number is PRJNA660118), accessed on 10 September 2020.

## Supplementary Materials

The following supporting information can be downloaded at https://www.mdpi.com/article/10.3390/ani14223200/s1, Table S1: A total of 100 polymorphism microsatellite loci in the Chinese mitten crab (*Eriocheir sinensis*).
